# The biodistribution of 5-[^18^F]fluoropyrazinamide in *Mycobacterium tuberculosis*-infected mice determined by positron emission tomography

**DOI:** 10.1371/journal.pone.0170871

**Published:** 2017-02-02

**Authors:** Zhuo Zhang, Alvaro A. Ordonez, Peter Smith-Jones, Hui Wang, Kayla R. Gogarty, Fereidoon Daryaee, Lauren E. Bambarger, Yong S. Chang, Sanjay K. Jain, Peter J. Tonge

**Affiliations:** 1 Institute for Chemical Biology & Drug Discovery, Department of Chemistry and Department of Radiology, Stony Brook University, Stony Brook, New York, United States of America; 2 Center for Infection and Inflammation Imaging Research, Center for Tuberculosis Research and Department of Pediatrics, Johns Hopkins University School of Medicine, Baltimore, Maryland, United States of America; 3 The Facility for Experimental Radiopharmaceutical Manufacturing, Department of Psychiatry, Stony Brook University, Stony Brook, New York, United States of America; Wayne State University, UNITED STATES

## Abstract

5-[^18^F]F-pyrazinamide (5-[^18^F]F-PZA), a radiotracer analog of the first-line tuberculosis drug pyrazinamide (PZA), was employed to determine the biodistribution of PZA using PET imaging and *ex vivo* analysis. 5-[^18^F]F-PZA was synthesized in 60 min using a halide exchange reaction. The overall decay-corrected yield of the reaction was 25% and average specific activity was 2.6 × 10^6^ kBq (70 mCi)/μmol. The biodistribution of 5-[^18^F]F-PZA was examined in a pulmonary *Mycobacterium tuberculosis* mouse model, where rapid distribution of the tracer to the lung, heart, liver, kidney, muscle, and brain was observed. The concentration of 5-[^18^F]F-PZA was not significantly different between infected and uninfected lung tissue. Biochemical and microbiological studies revealed substantial differences between 5-F-PZA and PZA. 5-F-PZA was not a substrate for pyrazinamidase, the bacterial enzyme that activates PZA, and the minimum inhibitory concentration for 5-F-PZA against *M*. *tuberculosis* was more than 100-fold higher than that for PZA.

## Introduction

PZA is a well-established anti-tuberculosis drug and a critical component of first- and second-line drug regimens against tuberculosis (TB), due to its unique ability to synergize with other TB drugs to shorten treatment duration [1, 2]. PZA is active against *Mycobacterium tuberculosis*, but not other mycobacterial or non-mycobacterial species [3, 4]. It is considered a pro-drug that is converted to the active form pyrazinoic acid (POA) by a mycobacterial amidase, pyrazinamidase (PZase), encoded by *pncA* [5]. While a majority of PZA-resistant isolates harbor *pncA* mutations [6], recent data suggest that host-mediated bioactivation of PZA also occurs [7]. Although the mechanism of PZA still remains to be fully elucidated [5, 8], the lack of definitive *in vitro* targets [4, 5, 9–11], and surprisingly high *in vitro* minimum inhibition concentration (MIC) against *M*. *tuberculosis* [12–14], suggests that PZA may also act through other mechanisms. Owing to the potent anti-inflammatory activity of the PZA analog nicotinamide [15], it has been hypothesized that the synergistic effects of PZA (or its active metabolite) may at least, in part, be due to anti-inflammatory effects.

Pyrazinamide is easily absorbed in the gastrointestinal track after oral administration and freely distributed to all parts of the body, including infected tissues. However, the kinetics and biodistribution in infected-tissues is not well understood. The distribution of both PZA and POA is similar in the caseum and cellular regions of resected granulomas of rabbits and humans [16]. Similar findings have also been reported in mice. However, the overall response to treatment with PZA was inferior in a subpopulation of the C3HeB/FeJ mouse model that develops necrotic granulomas, suggesting that some environmental factors within the lesions can affect the efficacy of PZA [17].

Thus, we sought to supplement these studies with a non-invasive method to study the tissue distribution of PZA with a radiolabeled analog, 5-[^18^F]F-PZA, in a pulmonary TB mouse model that develops necrotic lesions [18, 19], using positron emission tomography (PET). We synthesized 5-[^18^F]F-PZA via a halide exchange reaction and hypothesized that 5-F-PZA would recapitulate the biodistribution of PZA *in vivo*.

## Materials and methods

All protocols were approved by the Johns Hopkins and / or Stony Brook University Biosafety, Radiation Safety and Animal Care and Use Committees. All *in vitro* and animal experiments with *M*. *tuberculosis* were performed according to biosafety procedures in the animal biological safety level-3 (ABSL-3) facility at Johns Hopkins University School of Medicine.

### Synthesis of 5-F-PZA

**Fig 1:**
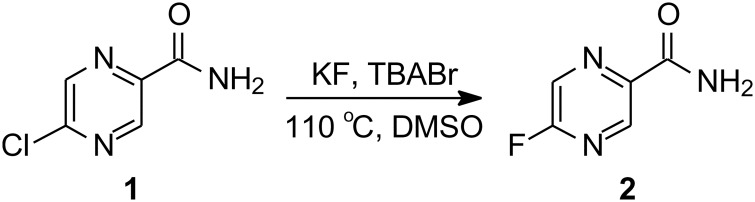
Synthesis of reference 5-F-PZA.

5-F-PZA was synthesized by halogen exchange from 5-Cl-PZA with potassium fluoride (KF), catalyzed by *tetra*-n-butylammonium bromide (TBABr) (Fig 1).

#### 5-Fluoropyrazinamide (2)

5-Chloropyrazinamide (**1**, 50 mg, 0.3173 mmol), potassium fluoride (KF) (110.43 mg, 1.90 mmol) and *tetra*-n-butylammonium bromide (TBABr) (20.46 mg, 0.064 mmol) were added to a 10 mL RBF and placed under vacuum for 1 hour to dry. After filling the RBF with N_2_, 10 mL of dry DMSO stored over molecular sieves was added to the RBF. The reaction mixture was heated to 110°C and refluxed for 30 min under N_2_. After the reaction was shown to be complete by TLC, the mixture was cooled to room temperature, diluted with iced water and then extracted with ethyl acetate. The organic layer was dried with MgSO_4_ and the solvent was evaporated by vacuum to yield crude product which was purified by CombiFlash chromatography using a silica column and petroleum ether and ethyl acetate as the eluent.

#### GC-EI Mass

Calculated for C_5_H_4_FN_3_O (M^•+^) 141.11, found 141.11. Two fragments peaks were also observed: M-CONH_2_^•+^ (C_4_H_2_FN_2_^•+^), m/z = 97.07 and M-CONH^+^ (C_4_H_2_FN_2_H^+^), m/z = 98.08.

^**1**^**H NMR** (400 MHz, CDCl_3_ and CD_3_OD): δ 9.00 (s, 1H), δ 8.36 (d, J = 7.8 Hz, 1H). ^**19**^**F NMR** (400 MHz, CDCl_3_ and CD_3_OD): δ 75.17 (d, J = 7.3 Hz). ^**13**^**C NMR** (500 MHz, CDCl_3_ and CD_3_OD): δ 164.72 (s, 1’-C), δ 161.56 (d, J = 256.5 Hz, 5’-C), δ 142.41 (d, J = 12.8 Hz, 3’-C), δ 142.27 (d, 2’-C) δ 131.78 (d, J = 38.7 Hz, 6’-C)

### Radiosynthesis of 5-[^18^F]F-PZA

**Fig 2:**
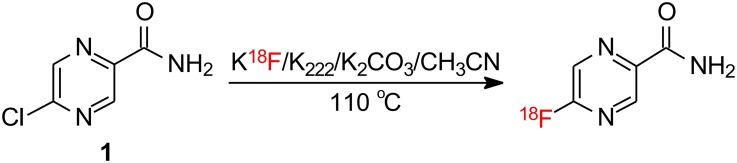
Radiosynthesis of 2-[^18^F]F-PZA.

5-[^18^F]F-PZA was synthesized via a halogen exchange reaction, similar to the method used to synthesize 5-F-PZA (Fig 2). 2960 MBq (80 mCi) of aqueous [^18^F]fluoride in ddH_2_O was purchased from PETNET Solutions Inc. The [^18^F]fluoride was placed in a vial containing Kryptofix 2.2.2 (5 mg, 13 μmol) and potassium carbonate (1 mg, 7 μmol) and the solution was then dried by azeotropic distillation. The solid residue was then re-solubilized with 0.2–0.3 mL of acetonitrile containing 1–2 mg 5-chloropyrazinamide (5-Cl-PZA). The reaction mixture was heated in a securely capped 2 mL reaction vial at 105°C for 8 min, and subsequently quenched with 0.8 mL water. The reaction mixture was filtered through a vented 0.22 um Millipore filter using a 1 mL syringe and then purified by HPLC with an isocratic mobile phase of 8% acetonitrile/92% 0.02 M aqueous ammonium acetate with 5% acetic acid (Phenomenex Luna PFP, 250 × 10, 5 μm, 4 mL/min). The radioactive product eluted at the same retention time as the 5-F-PZA standard (10 to 12 min). The solvent was removed by rotary evaporation. The residue was re-solubilized in sterile phosphate buffered saline and the pH of the solution was adjusted to 7.4 by the addition of 2M NaOH. The solution was filtered through an Acrodisc 13 mm syringe filter equipped with a 0.2 μm Supor membrane (Pall Corporation) into a sterile vial. Radiochemical purity was determined by reverse-phase analytical HPLC (Phenomenex PFP, 250 × 4.6, 5 μm, 1 mL/min, 8% acetonitrile/92% 0.02 M aqueous ammonium acetate with 5% acetic acid mobile phase).

### Pyrazinamidase assay

Wayne’s modified pyrazinamidase assay was performed as previously described [20] using a minor modification: the pyrazinamide concentrations were increased to enhance color change [21]. Briefly, Middlebrook 7H9 broth base (Becton Dickinson) was dissolved in water with glycerol and 1.5% agarose. PZA, 5-F-PZA, and 5-Cl-PZA were added to achieve a concentration of 400 μg/mL. Four mL aliquots of the melted agar were distributed into glass tubes and autoclaved. Subsequently, the tubes were inoculated with an actively growing culture of mycobacteria, and incubated for 4 days at 37°C. After the incubation period, 1 mL of ferrous ammonium sulfate (1%) was added to each tube and observed over 4 hours for the development of a red/orange/brown color change, indicating drug metabolism and susceptibility by the bacteria. The PZA-susceptible strain *M*. *tuberculosis* H37Rv was used as a positive control. The *M*. *tuberculosis* PZA-resistant strain (*pncA* mutation) and the naturally PZA-resistant *Mycobacterium bovis* Bacillus Calmette—Guérin (BCG) strain were used as negative controls. Each experimental condition was tested in four biological replicates.

### *In vitro* characterization of 5-[^18^F]F-PZA

The minimum inhibitory concentration (MIC) of 5-F-PZA against *M*. *tuberculosis* was determined by the agar dilution method, as previously described [22]. Briefly, Middlebrook 7H10 agar base (Becton Dickinson) was dissolved in deionized water with glycerol and acidified with monopotassium phosphate (KH_2_PO_4_). After autoclaving, the broth was supplemented with 10% fetal bovine serum and the pH was measured to be between 5.5 and 6. Varying concentrations of 5-F-PZA, ranging from 0.625 to 2000 μg/mL, were added to the media. A culture of *M*. *tuberculosis* H37Rv, in log phase growth, with an initial absorbance of 1.0 corresponding to approximately 10^8^ colony forming units (CFU)/mL was inoculated on duplicate plates for every concentration of 5-F-PZA. Plates were incubated at 37°C with 5% CO_2_ for 30 days. Broth containing no 5-F-PZA or PZA served as the negative control and PZA at various concentrations ranging from 0.625 to 100 μg/mL served as positive control. MIC was defined as the lowest concentration at which no visible growth was observed after 30 days of incubation.

*Staphylococcus aureus* [American Type Culture Collection (ATCC) 29213], *Escherichia coli* (ATCC 25922) and *Pseudomonas aeruginosa* (ATCC 10145) were grown at 37°C in Lysogeny Broth (LB) to an absorbance at 600 nm of 1.0. Frozen stocks of wild-type *M*. *tuberculosis* (H37Rv) were grown to mid-log phase in Middlebrook 7H9 broth supplemented with 10% oleic acid-albumin-dextrose-catalase (Difco) and 0.05% Tween 80 (Sigma-Aldrich). Accumulation assays were performed by incubating bacterial cultures with 20 kBq (0.5 μCi)/mL 5-[^18^F]F-PZA as described previously [23, 24]. Heat-killed (90°C for 30 min) bacteria were similarly incubated with each probe and served as a negative control. One mL samples of the bacteria cultures were taken at various time points, pelleted by centrifugation and washed three times with PBS in order to separate the bacteria cells from the culture broth. Subsequently, the radioactivity associated with the cells was measured using an automated γ counter (1282 Compugamma CS Universal gamma counter, LKB Wallac). For the final time point, 1 mL of the culture was taken and the radioactivity was measured without pelleting and washing. The radioactivity associated with the bacteria cells was divided by the radioactivity of the culture (bacteria cells and culture broth) to yield percent accumulation. Six replicates were used for each assay. In order to ensure there is no accumulation in mammalian cells, uptake assays were also performed using J774A.1 murine macrophage cells (ATCC). 2-[^18^F]F-2-deoxy-d-glucose (FDG), known to accumulate in mammalian cells, was used as a positive control. Tracer accumulation was measured as described above after 120 min of incubation.

### Animal experiments

#### *In vivo* aerosol infection

Four to six-week-old female C3HeB/FeJ (Jackson Laboratory) mice were aerosol infected with frozen stocks of *M*. *tuberculosis* H37Rv, using the Middlebrook Inhalation Exposure System (Glas-Col). Three mice were sacrificed using isoflurane (Henry Schein) overdose one day after infection to determine the number of bacilli implanted in the lungs and four infected mice from the same infection group that were not injected with tracer for imaging, were sacrificed to determine the bacillary burden at the time of imaging. The entire lungs from the sacrificed mice were homogenized in PBS, and then plated by serial dilution in triplicate onto Middlebrook 7H11 selective plates (Becton Dickinson). All plates were incubated at 37°C for 4 weeks before colonies were counted. A separate set of infected mice from the same aerosol infection group was used for imaging studies.

#### Bio-containment and anesthesia

Live *M*. *tuberculosis*-infected animals were imaged within a sealed bio-containment bed (Minerve) modified in-house to be compliant with biosafety level 3 (BSL3) containment, as described previously [25, 26]. Briefly, two 0.22 μm filters were used in series at both the inlet and the outlet of airflow to contain the bacteria within the bio-containment bed. A standard small animal anesthesia machine was used to deliver a mixture of isoflurane and oxygen to anesthetize during transport and imaging. A 30-gauge needle (BD Bioscience) was attached to polyethylene-10 tubing (Braintree Scientific) and inserted into the lateral tail vein of each mouse as a catheter for tracer delivery. Animals were sealed inside the bio-containment device in the ABSL-3 facility and the external surfaces of the bio-containment device were decontaminated with a 1:256 LpH:water solution (Steris) followed by 70% ethanol and transported to the imaging suite.

#### 5-[^18^F]F-PZA-PET/CT imaging and biodistribution

C3HeB/FeJ mice were weighed, injected with 7.4 MBq (0.2 mCi) 5-[^18^F]F-PZA via the tail vein catheter while anesthetized in the PET/CT scanner. Dynamic PET imaging was performed using the Mosaic HP Small Animal Imager (Philips) for 60 min with 5 two min frames followed by 10 five min frames. Three *M*. *tuberculosis*-infected and three uninfected, healthy controls were scanned two at a time; a healthy control mouse was scanned simultaneously with each infected mouse. Computed tomography (CT) scans were immediately performed subsequent to PET imaging using the NanoSPECT/CT (Bioscan) *in vivo* animal imager. PET data were reconstructed and co-registered with CT images. The images were automatically co-registered and presented using VivoQuant 2.5 (Invicro) with a resulting 1:1 correspondence. Each animal CT had two spherical (3 mm diameter) regions of interest (ROI) drawn manually in the lung fields making sure not to overlap the PET-active liver while single ROIs were outlined for liver, left ventricle of the heart (as a surrogate for blood), and bone (thoracic vertebrae). Percentage injected dose per cubic centimeter (%ID/cc) was calculated using Amide version 1.0.4 (http://www.amide.sourceforge.net). For each group, the mean lung 5-[^18^F]F-PZA-PET activity at each time-point was calculated by averaging the normalized lung %ID/cc of all the ROIs in that group. Mean lung 5-[^18^F]F-PZA-PET activity at each time-point was also calculated similarly for uninfected animals used as controls and imaged at the same time. After imaging, all mice were sacrificed by isoflurane overdose to collect tissues for direct γ counting (biodistribution). The weight of each resected tissue was recorded prior to gamma counting. The biodistribution data are presented as percent injected dose per gram of tissue (%ID/g).

#### CD-1 mouse liver microsomal stability of 5-F-PZA

Test compounds were weighed and dissolved in dimethyl sulfoxide (DMSO) to give 10 mM stock solutions. The stock solutions were diluted to 500 μM with a mixture of methanol and H_2_O (1:1), and the final concentrations of DMSO and methanol were equal to, or less than, 0.1%. Liver microsome incubations were conducted in duplicate in 96—well plates. Each well contained 40 μL of 0.1 M potassium phosphate buffer (pH 7.4), 2.5 mM MgCl_2_, 0.625 mg/mL mouse liver microsomes, and test compound (1.25 μM) or positive control. After a 5 min pre-incubation at 37°C, 10 μL of 5.0 mM NADPH in 0.1 M potassium phosphate buffer was added to initiate the enzymatic reaction. The final concentrations were 0.1M potassium phosphate (pH 7.4), 1.0 mM NADPH, 2.0 mM MgCl_2_, 0.5 mg/mL mouse liver microsomes, and test compound (1.0 μM) or positive control (1.0 μM). Reactions were terminated at various time points (0, 5, 10, 20, 40 min) by adding 100 μL of ice-cold acetonitrile containing internal standard. A parallel incubation was performed using 0.1 M potassium phosphate (pH 7.4) instead of NADPH as the negative control, and the reaction was terminated 40 min after incubation at 37°C. Compound quantitation was performed on a Shimadzu liquid chromatographic system coupled with an API4000 mass spectrometer equipped with TurboIonSpray (ESI) Interface (Applied Biosystems).

#### *Ex vivo* metabolite analysis of 5-[^18^F]F-PZA

Mouse livers were harvested one day before the experiment and stored at -80°C. The livers were placed in phosphate buffer and then homogenized on ice. On the day of the experiment 50 μL of 5-[^18^F]F-PZA (740 kBq, 20 μCi) in saline (with 1% BSA), along with 50 μL of raw liver homogenate, was added to 900 μL phosphate buffer in a 15 mL Eppendorf tube. The mixture was incubated in an orbital shaker at room temperature for 2, 30, 60, 90, 120 min, respectively. A 100 μL aliquot was removed from each sample at the latter time points. The aliquot was centrifuged and then a 50 μL aliquot of the supernatant was counted, along with appropriate standards, using a γ counter (Wizard 2480, Perkin Elmer). Subsequently, a 20 μL aliquot of the supernatant was spotted on a TLC plate which was developed using 80% ethyl acetate in hexane and analyzed using a radio TLC scanner (AR-2000, Eckert & Ziegler).

## Results

**Fig 3:**
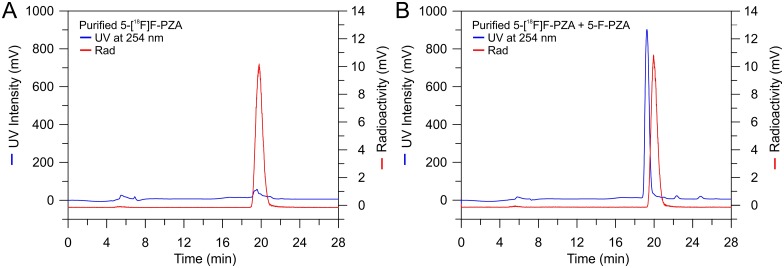
Analytical HPLC chromatography of 5-[^18^F]F-PZA. (A) HPLC chromatography of purified 5-[^18^F]F-PZA. The blue trace is the UV absorption of the eluent at 254 nm from an injection of purified 5-[^18^F]F-PZA. The red trace is the radioactive signal of the injected 5-[^18^F]F-PZA. (B) Co-injection of standard 5-F-PZA with purified 5-[^18^F]F-PZA. The blue trace is the UV absorption of the eluent at 254 nm of purified 5-[^18^F]F-PZA spiked with cold standard 5-F-PZA. The red trace is the radioactive signal of the purified 5-[^18^F]F-PZA spiked with cold standard 5-F-PZA.

5-F-PZA and 5-[^18^F]F-PZA were synthesized by halogen exchange from 5-Cl-PZA. 5-[^18^F]F-PZA was synthesized in approximately 60 min from [^18^F]fluoride in an overall decay-corrected yield of 25% and with an average specific activity of 2.6 × 10^6^ kBq (70 mCi)/μmol. Analytical HPLC demonstrated that after purification there was a single peak on the radioactive HPLC chromatography, suggesting that the final tracer is pure (Fig 3A). In addition, co-injection of 5-F-PZA with the purified tracer resulted in the appearance of an UV absorption peak with the same retention time as that detected using the inline radioactivity detector (Fig 3B). Analytical HPLC thus verified that the radiosynthesis was successful and the radioactive peak that was collected contained 5-[^18^F]F-PZA.

The Wayne’s modified pyrazinamidase assay was used to determine if 5-F-PZA was recognized and metabolized by the *M*. *tuberculosis* pyrazinamidase enzyme that converts PZA to POA. While PZA and 5-Cl-PZA were metabolized by this enzyme, 5-F-PZA was not (Table 1). The MIC of PZA in acidic agar (pH 5.5 to 6) was determined to be 20 μg/mL, however 5-F-PZA did not inhibit bacterial growth up to a concentration of 2 mg/mL. *In vitro* accumulation of 5-[^18^F]F-PZA after 120 min of incubation was 0.094%±0.032 in *M*. *tuberculosis*, 0.123±0.014 in *S*. *aureus*, 0.055±0.005 in *E*. *coli* and 0.043±0.026 in *P*. *aeruginosa*. These results were similar to the accumulation of 5-[^18^F]F-PZA in J774A.1 murine macrophage cells (0.057±0.019). There was no uptake in heat-killed control cultures.

**Table 1:** Wayne’s modified pyrazinamidase assay.

	PZA	5-Cl-PZA	5-F-PZA
*M*. *tuberculosis* (H37Rv)	++	+	-
*M*. *tuberculosis* (PZA resistant)	-	+	-
*M*. *bovis* (BCG)	-	-	-

**Fig 4:**
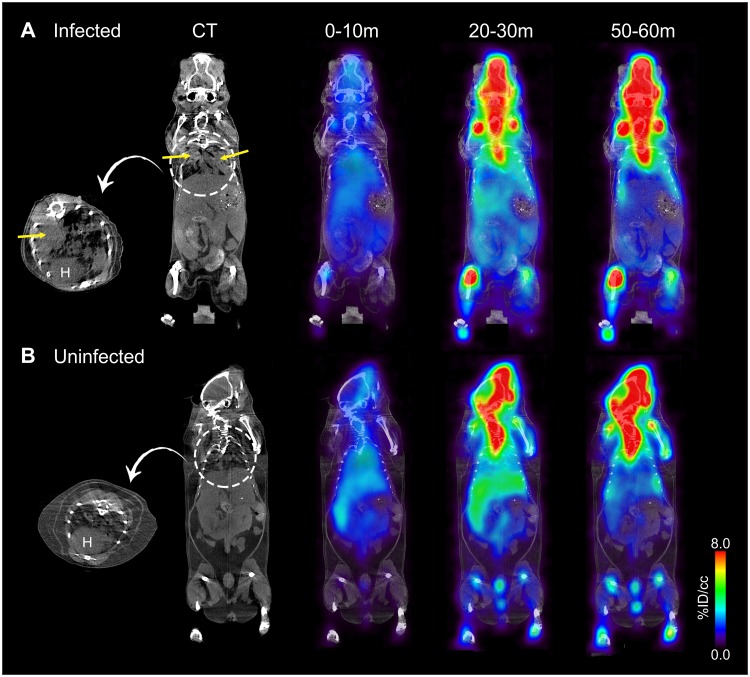
Dynamic PET/CT imaging of 5-[^18^F]F-PZA in infected and uninfected mice. (A) Dynamic PET/CT images of a representative *M*. *tuberculosis*-infected mouse. Lung consolidations (yellow arrows) can be observed in the transverse and coronal CT sections. PET/CT images 0 to 10 min, 20 to 30 min and 50 to 60 min post tracer administration. (B) Dynamic PET/CT images of a representative uninfected control mouse. The images showed in the figure are representatives of 3 animals. H = heart.

**Fig 5:**
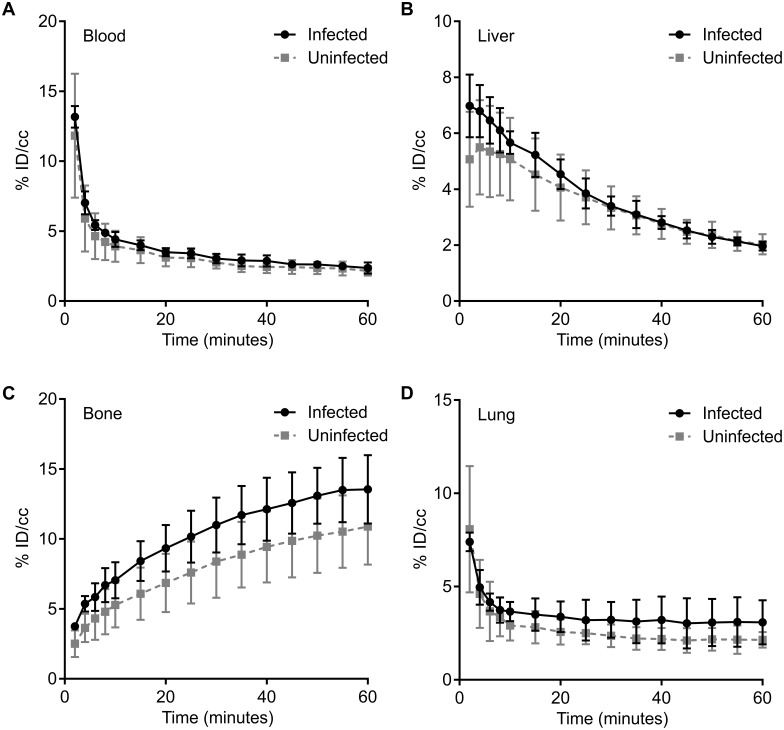
Organ compartment pharmacokinetics of 5-[^18^F]F-PZA. After a single intravenous injection of 5-[^18^F]F-PZA, a dynamic PET was acquired over 60 min and biodistribution data was calculated for blood (A), liver (B), bone (C) and lungs (D). No significant difference between the concentration of 5-[^18^F]F-PZA in infected and uninfected animal tissues was observed. Data are represented as mean and standard deviation of the percent injected dose per volume (%ID/cc). Three animals were used for each group.

**Fig 6:**
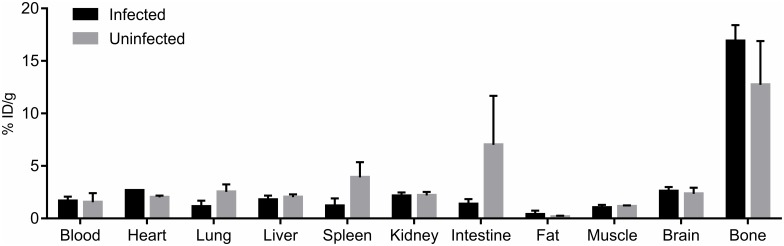
*Ex vivo* tissue biodistribution of 5-[^18^F]F-PZA. *M*. *tuberculosis*-infected mice and the age-matched uninfected controls were sacrificed after PET imaging to collect tissues for direct γ counting. The concentration of 5-[^18^F]F-PZA was similar between infected and uninfected mice in blood, heart, liver, kidney, muscle, and brain, while there was a higher activity in uninfected lung and spleen tissues compared to the infected group. The tissue weight of infected lung and spleens were significantly higher compared to uninfected controls. Data is presented as the mean and standard deviation of the percent injected dose per gram of tissue (%ID/g). Three animals were used for each group.

The biodistribution of 5-[^18^F]F-PZA was imaged using PET in *M*. *tuberculosis*-infected mice 10 weeks after aerosol infection. The pulmonary bacterial burden at the time of imaging was log_10_ 8.1 ± 0.7 CFU/mL. Co-registered 5-[^18^F]F-PZA PET/CT images of infected and uninfected animals are shown in Fig 4. The concentration of 5-[^18^F]F-PZA in blood, liver, bone, and lungs, determined as %ID/cc throughout 60 min post-injection, was similar in infected and uninfected mice (Fig 5). Subsequently, the animals were sacrificed and tissues harvested for direct γ counting (Fig 6). The *ex-vivo* concentration of 5-[^18^F]F-PZA was similar between infected and uninfected controls in the blood, heart, liver, kidney, muscle, and brain, while there was a higher activity in uninfected lung tissue compared to infected lungs. The small difference in lung radioactivity may be due to the difference in lung size; the average weight of the infected lungs was 0.76±0.05 g, which is significantly larger when compared to 0.20±0.04 g in uninfected controls (*P*<0.001).

**Fig 7:**
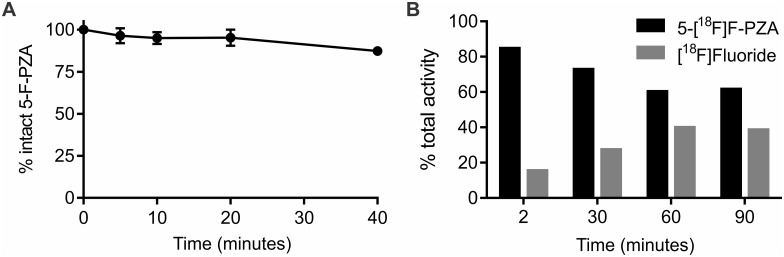
5-[^18^F]F-PZA stability and defluorination. (A) Percentage of intact 5-F-PZA after incubation with mouse liver microsomes over 40 min. The amount of intact 5-F-PZA available at each time point was determined by LC-MS and is presented as percentage of total 5-F-PZA added. (B) Percentage of intact 5-[^18^F]F-PZA and [^18^F]fluoride after incubation of 5-[^18^F]F-PZA with mouse liver homogenates over 90 min. The amount of 5-[^18^F]F-PZA and [^18^F]fluoride was determined by using radioactive TLC. Data are presented as the percentage of total activity on the TLC plates.

The high bone uptake suggested the presence of free circulating [^18^F]fluoride, which may be a result of the defluorination of 5-[^18^F]F-PZA *in vivo*. To assess the metabolic stability of 5-[^18^F]F-PZA, 5-F-PZA was incubated with CD-1 mouse liver microsomes and approximately 20% of the initial concentration of 5-F-PZA incubated was determined to be metabolized after 40 min (Fig 7A). Subsequently, TLC-based *ex vivo* metabolite analysis demonstrated that 5-[^18^F]F-PZA is rapidly defluorinated by mouse liver homogenates (Fig 7B). Defluorination could be detected after only 2 min incubation and ~25% of the total activity was defluorinated by 30 min. The percent defluorination increased to ~40% by 60 min and remained constant afterwards.

## Discussion

Molecular imaging provides noninvasive and rapid evaluation of disease processes. Dynamic PET can provide multi-compartment analyses at multiple time points, allowing the characterization of radiolabeled drug concentrations in multiple tissues simultaneously. Previous studies with whole-body PET bioimaging in murine models of TB have provided valuable information about the pharmacokinetics of the first-line TB drugs isoniazid (2-[^18^F]fluoroisonicotinic acid hydrazide, [26]) and rifampin (^11^C-rifampin, [27]).

5-[^18^F]F-PZA was successfully synthesized from 5-Cl-PZA by halogen exchange. The SNAr-based halogen exchange method used often involves a more nucleophilic fluorine source such as *tetra*-n-butylammonium fluoride rather than potassium fluoride in combination with an electron-deficient aromatic ring [28, 29]. In the case of 5-Cl-PZA we reasoned that the inductive electron withdrawing effect of the nitrogen adjacent to the 5’ position together with the conjugating ability of the nitrogen would collectively assist the SNAr substitution reaction. This was found to be the case, and the radiosynthesis of 5-[^18^F]F-PZA was accomplished in a one-step reaction in a total synthesis time of 60 min. This method offers a reasonably high yield with a typical decay-corrected yield of 25%.

The objective of this study was to determine the tissue biodistribution of 5-[^18^F]F-PZA, particularly in *M*. *tuberculosis*-infected mouse lungs, in order to reveal further information on tissue biodistribution of PZA. The feasibility of this objective naturally depended on the chemical and biological similarity of 5-F-PZA to PZA. 5-F-PZA exhibited poor *in vitro* activity (MIC of >2000 μg/mL in *M*. *tuberculosis* H37Rv), much higher (100-fold) than the MIC of PZA in the same strain (20 μg/mL). As expected, PZA was hydrolyzed by a PZA-susceptible strain of *M*. *tuberculosis* (H37Rv), but not by a PZA-resistant strain (*pncA* mutant) or the naturally PZA-resistant *M*. *bovis*. No hydrolysis was observed for 5-F-PZA for both H37Rv and PZA-resistant strains. 5-Cl-PZA did show a lower extent of hydrolysis in H37Rv, and a similar extent of hydrolysis was also seen in the PZA-resistant strain. Previous experiments have reported that 5-Cl-PZA has *in vitro* activity against PZA-resistant *M*. *tuberculosis* isolates, suggesting that 5-Cl-PZA does not require activation by the mycobacterial PZase [14, 30]. Subsequent animal experiments showed lack of activity of 5-Cl-PZA in *M*. *tuberculosis*-infected mice [31]. Our findings indicate that 5-F-PZA is not a substrate for the *M*. *tuberculosis* PZase enzyme. However, given the recent findings on a diverse range of possible targets for PZA [10, 32, 33], we cannot rule out other mechanisms where 5-F-PZA can have some activity against *M*. *tuberculosis* within the host, including host-mediated conversion into 5-F-POA [7].

PET imaging demonstrated that 5-[^18^F]F-PZA rapidly distributed to various organs after intravenous administration. However, the tracer was rapidly eliminated from all major organs and no significant difference in accumulation observed between the infected and healthy tissues in mice. PET imaging also revealed significant activity in the bones 10 min after tracer administration, and especially at 30 min post-injection. High bone activity was also seen in post-mortem analysis. All of these observations suggested rapid defluorination of 5-[^18^F]F-PZA *in vivo*. Metabolic studies indicated that 5-F-PZA was not stable in the presence of liver microsomes, and specific defluorination of 5-F-PZA was confirmed by *ex vivo* metabolite analysis. The rapid and extensive defluorination of 5-[^18^F]F-PZA likely hinders the tracer from accumulating specifically in infected tissues. Previous PET imaging in the same TB mouse model demonstrated that [^18^F]fluoride did not distribute to the heart, lungs, liver or brain of mice [34], while distribution to these organs was observed for 5-[^18^F]F-PZA.

## Conclusion

In conclusion, we present a successful synthesis of radiolabeled 5-[^18^F]F-PZA. However, 5-F-PZA was not converted into 5-F-POA by *M*. *tuberculosis* PZase, but the possibility of other host-mediated activation pathways has not been ruled out. 5-[^18^F]F-PZA distributes to various peripheral organs rapidly after intravenous administration but with no significant accumulation in any of these organs. The fast elimination of 5-[^18^F]F-PZA in combination with the lack of sustained and specific interaction with mycobacterial targets, and the defluorination observed in mice might limit the imaging potential of 5-[^18^F]F-PZA for TB.

## References

[1] Dawson R, Van Niekerk C, Erondu N, Ginsberg A, editors. P-931a—Pyrazinamide Increases the Early Bactericidal Activity of TMC207 and PA-824 in Patients with Newly Diagnosed, Smear-positive Pulmonary Tuberculosis. ICAAC; 2011 Sep 18, 2011; Chicago, IL.

[2] American Thoracic Society. Medical Section of the American Lung Association: Treatment of tuberculosis and tuberculosis infection in adults and children. The American review of respiratory disease. 1986;134(2):355–63. Epub 1986/08/01. 3527010 10.1164/arrd.1986.134.2.355

[3] Sun Z, Scorpio A, Zhang Y. The pncA gene from naturally pyrazinamide-resistant Mycobacterium avium encodes pyrazinamidase and confers pyrazinamide susceptibility to resistant M. tuberculosis complex organisms. Microbiology (Reading, England). 1997;143 (Pt 10):3367–73. Epub 1997/11/14. 10.1099/00221287-143-10-3367 9353938

[4] Boshoff HI, Mizrahi V, Barry CE 3rd. Effects of pyrazinamide on fatty acid synthesis by whole mycobacterial cells and purified fatty acid synthase I. Journal of bacteriology. 2002;184(8):2167–72. Epub 2002/03/27. 10.1128/JB.184.8.2167-2172.2002 11914348 PMC134955

[5] Zhang Y, Mitchison D. The curious characteristics of pyrazinamide: a review. The international journal of tuberculosis and lung disease: the official journal of the International Union against Tuberculosis and Lung Disease. 2003;7(1):6–21. 12701830

[6] Chang KC, Yew WW, Zhang Y. Pyrazinamide susceptibility testing in Mycobacterium tuberculosis: a systematic review with meta-analyses. Antimicrobial agents and chemotherapy. 2011;55(10):4499–505. Epub 2011/07/20. 10.1128/AAC.00630-11 21768515 PMC3186960

[7] Via LE, Savic R, Weiner DM, Zimmerman MD, Prideaux B, Irwin SM, et al Host-Mediated Bioactivation of Pyrazinamide: Implications for Efficacy, Resistance, and Therapeutic Alternatives. ACS infectious diseases. 2015;1(5):203–14. Epub 2015/06/19. 10.1021/id500028m 26086040 PMC4467917

[8] Singh P, Mishra AK, Malonia SK, Chauhan DS, Sharma VD, Venkatesan K, et al The paradox of pyrazinamide: an update on the molecular mechanisms of pyrazinamide resistance in Mycobacteria. The Journal of communicable diseases. 2006;38(3):288–98. Epub 2007/03/22. 17373362

[9] Pyrazinamide. Tuberculosis (Edinburgh, Scotland). 2008;88(2):141–4. Epub 2008/05/20. 10.1016/S1472-9792(08)70021-0 18486055

[10] Zimhony O, Cox JS, Welch JT, Vilcheze C, Jacobs WR Jr. Pyrazinamide inhibits the eukaryotic-like fatty acid synthetase I (FASI) of Mycobacterium tuberculosis. Nature medicine. 2000;6(9):1043–7. Epub 2000/09/06. 10.1038/79558 10973326

[11] Shi W, Zhang X, Jiang X, Yuan H, Lee JS, Barry CE 3rd, et al Pyrazinamide inhibits trans-translation in Mycobacterium tuberculosis. Science (New York, NY). 2011;333(6049):1630–2. Epub 2011/08/13. 10.1126/science.1208813 PMC3502614 21835980

[12] Scorpio A, Zhang Y. Mutations in pncA, a gene encoding pyrazinamidase/nicotinamidase, cause resistance to the antituberculous drug pyrazinamide in tubercle bacillus. Nature medicine. 1996;2(6):662–7. Epub 1996/06/01. 8640557 10.1038/nm0696-662

[13] Baughn AD, Deng J, Vilcheze C, Riestra A, Welch JT, Jacobs WR Jr., et al Mutually exclusive genotypes for pyrazinamide and 5-chloropyrazinamide resistance reveal a potential resistance-proofing strategy. Antimicrobial agents and chemotherapy. 2010;54(12):5323–8. Epub 2010/09/30. 10.1128/AAC.00529-10 20876380 PMC2981270

[14] Cynamon MH, Speirs RJ, Welch JT. In vitro antimycobacterial activity of 5-chloropyrazinamide. Antimicrobial agents and chemotherapy. 1998;42(2):462–3. Epub 1998/04/04. 9527809 10.1128/aac.42.2.462 PMC105437

[15] Ungerstedt JS, Blomback M, Soderstrom T. Nicotinamide is a potent inhibitor of proinflammatory cytokines. Clin Exp Immunol. 2003;131(1):48–52. Epub 2003/01/10. 10.1046/j.1365-2249.2003.02031.x 12519385 PMC1808598

[16] Prideaux B, Via LE, Zimmerman MD, Eum S, Sarathy J, O'Brien P, et al The association between sterilizing activity and drug distribution into tuberculosis lesions. Nature medicine. 2015;21(10):1223–7. Epub 2015/09/08. 10.1038/nm.3937 26343800 PMC4598290

[17] Irwin SM, Prideaux B, Lyon ER, Zimmerman MD, Brooks EJ, Schrupp CA, et al Bedaquiline and Pyrazinamide Treatment Responses Are Affected by Pulmonary Lesion Heterogeneity in Mycobacterium tuberculosis Infected C3HeB/FeJ Mice. ACS infectious diseases. 2016;2(4):251–67. 10.1021/acsinfecdis.5b00127 27227164 PMC4874602

[18] Harper J, Skerry C, Davis SL, Tasneen R, Weir M, Kramnik I, et al Mouse model of necrotic tuberculosis granulomas develops hypoxic lesions. J Infect Dis. 2012;205(4):595–602. 10.1093/infdis/jir786 22198962 PMC3266133

[19] Ordonez AA, Tasneen R, Pokkali S, Xu Z, Converse PJ, Klunk MH, et al Mouse model of pulmonary cavitary tuberculosis and expression of matrix metalloproteinase-9. Disease Models and Mechanisms. 2016;9(7):779–88. 10.1242/dmm.025643 27482816 PMC4958312

[20] Wayne LG. Simple pyrazinamidase and urease tests for routine identification of mycobacteria. The American review of respiratory disease. 1974;109(1):147–51. Epub 1974/01/01. 4203284 10.1164/arrd.1974.109.1.147

[21] Singh P, Wesley C, Jadaun GP, Malonia SK, Das R, Upadhyay P, et al Comparative evaluation of Lowenstein-Jensen proportion method, BacT/ALERT 3D system, and enzymatic pyrazinamidase assay for pyrazinamide susceptibility testing of Mycobacterium tuberculosis. Journal of clinical microbiology. 2007;45(1):76–80. Epub 2006/11/10. 10.1128/JCM.00951-06 17093022 PMC1828947

[22] Heifets L, Sanchez T. New agar medium for testing susceptibility of Mycobacterium tuberculosis to pyrazinamide. Journal of clinical microbiology. 2000;38(4):1498–501. 10747133 10.1128/jcm.38.4.1498-1501.2000 PMC86474

[23] Ordonez AA, Weinstein EA, Bambarger LE, Saini V, Chang YS, DeMarco VP, et al A Systematic Approach for Developing Bacteria-Specific Imaging Tracers. J Nucl Med. 2016. 10.2967/jnumed.116.181792 PMC5209639 27635025

[24] Weinstein EA, Ordonez AA, DeMarco VP, Murawski AM, Pokkali S, MacDonald EM, et al Imaging Enterobacteriaceae infection in vivo with 18F-fluorodeoxysorbitol positron emission tomography. Sci Transl Med. 2014;6(259):259ra146 10.1126/scitranslmed.3009815 25338757 PMC4327834

[25] Davis SL, Nuermberger EL, Um PK, Vidal C, Jedynak B, Pomper MG, et al Noninvasive pulmonary [18F]-2-fluoro-deoxy-D-glucose positron emission tomography correlates with bactericidal activity of tuberculosis drug treatment. Antimicrobial agents and chemotherapy. 2009;53(11):4879–84. 10.1128/AAC.00789-09 19738022 PMC2772305

[26] Weinstein EA, Liu L, Ordonez AA, Wang H, Hooker JM, Tonge PJ, et al Noninvasive determination of 2-[18F]-fluoroisonicotinic acid hydrazide pharmacokinetics by positron emission tomography in Mycobacterium tuberculosis-infected mice. Antimicrobial agents and chemotherapy. 2012;56(12):6284–90. 10.1128/AAC.01644-12 23006755 PMC3497161

[27] DeMarco VP, Ordonez AA, Klunk M, Prideaux B, Wang H, Zhuo Z, et al Determination of [11C]rifampin pharmacokinetics within Mycobacterium tuberculosis-infected mice by using dynamic positron emission tomography bioimaging. Antimicrobial agents and chemotherapy. 2015;59(9):5768–74. 10.1128/AAC.01146-15 26169396 PMC4538528

[28] Furuya T, Kuttruff CA, Ritter T. Carbon-fluorine bond formation. Current opinion in drug discovery & development. 2008;11(6):803–19. Epub 2008/10/24. 18946845

[29] Preshlock S, Tredwell M, Gouverneur V. (18)F-Labeling of Arenes and Heteroarenes for Applications in Positron Emission Tomography. Chemical reviews. 2016;116(2):719–66. Epub 2016/01/12. 10.1021/acs.chemrev.5b00493 26751274

[30] Cynamon MH, Gimi R, Gyenes F, Sharpe CA, Bergmann KE, Han HJ, et al Pyrazinoic acid esters with broad spectrum in vitro antimycobacterial activity. Journal of medicinal chemistry. 1995;38(20):3902–7. 7562923 10.1021/jm00020a003

[31] Ahmad Z, Tyagi S, Minkowsk A, Almeida D, Nuermberger EL, Peck KM, et al Activity of 5-chloro-pyrazinamide in mice infected with Mycobacterium tuberculosis or Mycobacterium bovis. The Indian journal of medical research. 2012;136(5):808–14. 23287128 PMC3573602

[32] Manca C, Koo MS, Peixoto B, Fallows D, Kaplan G, Subbian S. Host targeted activity of pyrazinamide in Mycobacterium tuberculosis infection. PloS one. 2013;8(8):e74082 10.1371/journal.pone.0074082 24015316 PMC3755974

[33] Zhang S, Chen J, Shi W, Liu W, Zhang W, Zhang Y. Mutations in panD encoding aspartate decarboxylase are associated with pyrazinamide resistance in Mycobacterium tuberculosis. Emerging microbes & infections. 2013;2(6):e34. 26038471 10.1038/emi.2013.38 PMC3697303

[34] Ordonez AA, DeMarco VP, Klunk MH, Pokkali S, Jain SK. Imaging Chronic Tuberculous Lesions Using Sodium [(18)F]Fluoride Positron Emission Tomography in Mice. Molecular imaging and biology: MIB: the official publication of the Academy of Molecular Imaging. 2015;17(5):609–14. Epub 2015/03/10. 25750032 10.1007/s11307-015-0836-6 PMC4561601

